# Effect of Metal Foam Insert Configurations on Flow Boiling Heat Transfer and Pressure Drop in a Rectangular Channel

**DOI:** 10.3390/ma14164617

**Published:** 2021-08-17

**Authors:** Sanghyun Nam, Dae Yeon Kim, Youngwoo Kim, Kyung Chun Kim

**Affiliations:** School of Mechanical Engineering, Pusan National University, Busan 46241, Korea; namsh@wonik.com (S.N.); dykim77@pusan.ac.kr (D.Y.K.); ywkim@pusan.ac.kr (Y.K.)

**Keywords:** open-cell metal foam, insert configuration, flow boiling, heat transfer, pressure drop, goodness factor

## Abstract

Heat transfer under flow boiling is better in a rectangular channel filled with open-cell metal foam than in an empty channel, but the high pressure drop is a drawback of the empty channel method. In this study, various types of metal foam insert configurations were tested to reduce the pressure drop while maintaining high heat transfer. Specifically, we measured the boiling heat transfer and pressure drop of a two-phase vertical upward flow of R245fa inside a channel. To measure the pressure and temperature differences of the metal foam, differential pressure transducers and T-type thermocouples were used at both ends of the test section. While the saturation pressure was kept constant at 5.9 bar, the steam quality at the inlet of the test section was changed from 0.05 to 0.99. The channel height, moreover, was 3 mm, and the mass flux ranged from 133 to 300 kg/m^2^s. The two-phase flow characteristics were observed through a high-speed visualization experiment. Heat transfer tended to increase with the mean vapor quality, and, as expected, the fully filled metal foam channel offered the highest thermal performance. The streamwise insert pattern model had the lowest heat transfer at a low mass flux. However, at a higher mass flux, the three different insert models presented almost the same heat transfer coefficients. We found that the streamwise pattern model had a very low pressure drop compared to that of the spanwise pattern models. The goodness factors of the flow area and the core volume of the streamwise patterned model were higher than those of the full-filled metal foam channel.

## 1. Introduction

Open metal foam has a typical porous structure, with many randomly connected internal pathways and high porosity. The thermal performance of the heat exchanger can be improved by using metal foam inserts with a high surface area to volume ratio (790–2740 m^2^/m^3^) and high porosity (>90%). Several groups have published experimental research on metal foam heat exchangers [1,2,3,4,5,6], and, to date, many studies have been conducted on the convective heat transfer of single-phase liquids or gases using small heat exchangers and metal foams [7,8,9,10,11,12,13,14,15,16,17,18,19].

The phase change inside a channel filled with metal foam represents a very interesting heat transfer mechanism. A stochastic metal foam structure facilitates greater nucleation, leads to bubble break-ups, and inhibits bubble growth. The metal foam structure, moreover, makes the flow field more uniform and improves heat transfer performance. Several interesting and non-traditional heat-transfer boiling phenomena have been observed in such structures; these properties may differ from those of normal channel flows [20].

The first flow-pattern map for a two-phase gas–liquid flow was proposed by Baker [21] based on observations from the flow of bottles of gas and condensate from petroleum products in horizontal pipes. Since then, more flow pattern maps have been developed. The most commonly accepted of these maps are those of Mandhane et al. [22] for horizontal flow and Hewitt and Roberts [23] for vertical flow channels. However, previous flow pattern maps have limitations because their observations were performed under a narrow range of experimental conditions.

Topin et al. [24] conducted a convective boiling heat transfer experiment using n-pentane in a rectangular channel (10 × 50 × 100 mm^3^) filled with 40 PPI (pores per inch) copper foam. Zhao et al. [25] studied the flow boiling heat transfer of R134a refrigerant flowing through a horizontal tube containing copper metal foam. Their experiments were performed at mass flow rates of 26–106 kg/m^2^s and saturation pressures of 3.5 and 6 bar, controlled by an electric heater. Li and Leong [26] studied the flow boiling properties of FC-72 with flowing water inside aluminum metal foam. The authors experimentally and numerically studied the heat transfer process before nuclear boiling and at the onset of nuclear boiling, as well as the hysteresis effect.

Flow pattern mapping is currently a popular method for analyzing and characterizing two-phase boiling flow results. Zhao and Lu [25,27] presented a flow pattern map based on experimental results for a channel featuring a 26 mm inner diameter with 20 and 40 PPI copper foams. The flow patterns were determined by the patterns of local temperature fluctuations, which were mainly divided into two types. One type of pattern was an annular flow in which liquid film occupied the entire channel surface, and the other was a wavy flow in which the liquid partially wetted the channel walls. As the mass flow rate and vapor quality changed, the flow patterns divided into five flow regimes: stratified wavy, slug/wavy, plug/slug, plug, and annular.

Li et al. [28] quantitatively and qualitatively analyzed the flow boiling of R141b inside a channel filled with metal foam by visualizing the channel flow and investigating the boiling heat transfer performance. The flow patterns had a strong correlation with the heat transfer coefficient (HTC), and the dry-out phenomenon significantly reduced HTC. HTC also increased at the beginning of nucleation boiling and decreased at certain vapor quality points. This phenomenon was delayed as the mass flux decreased.

Kim et al. [29] presented the experimental results for the boiling heat transfer of R245fa in a channel filled with 18 PPI copper metal foam. In the planar channel without metal foam, three different flow patterns were observed. Different boiling flow patterns were also observed in the metallic porous media channels, and their properties were very different from those of the planar channels. The spanwise distribution of the two-phase flow was somewhat flat in the metal foam. With low vapor quality, HTC increased with foaming in the test section. For the churn-annular flow regime, HTC increased smoothly.

Studies of the thermal and fluid dynamics of metallic porous media have been carried out, but only studies of channels fully filled with porous structures have been reported. Metal structures with porosity greatly improve thermal transfer performance but involve strong pressure loss under fluid flow inside the channel with porous structures. As noted in previous studies, boiling flows feature certain flow patterns depending on the quality of the steam, and the rates of their mass flow and flow are generally uneven. Effective heat transfer media can be developed by manipulating the array patterns of porous structures, thereby optimizing the geometry for the purpose of inducing uniform flow and reducing overall pressure loss.

To improve thermal efficiency, porous media partially filled with patterned configurations should be investigated. Thus, in this study, an experimental investigation was conducted on the heat and fluid flow characteristics of different types of patterned configuration models with porous media. The boiling flows of three types of patterned models were examined and compared in a rectangular channel fully filled with copper porous media.

## 2. Experimental Methods

### 2.1. Metallic Porous Foam Structure

**Figure 1:**
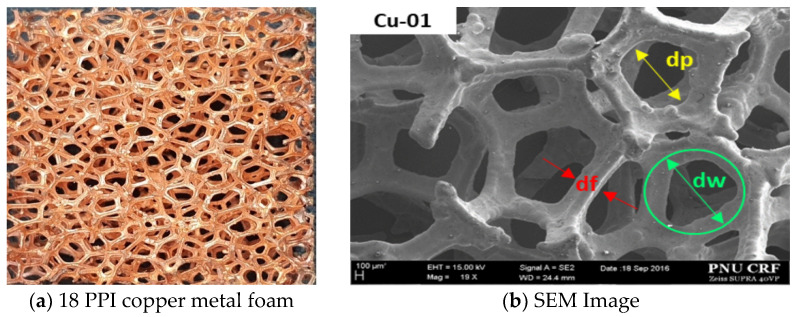
Image (**a**) and SEM image (**b**) of 18 PPI copper metal foam.

The metallic porous foams were produced in a local factory by Foam Tech Global, Inc. The metallic porous media were made with copper at 18 PPI and nickel at 24 PPI. As shown in Figure 1, 50 data points were averaged by the type of structure using SEM images to measure the diameters of the windows, fibers, and pores in 16 specimens. Table 1 shows the geometric parameters of the specimens. The porosity of all specimens was measured in a non-invasive manner based on perfect gas law properties using the method described by Beranek [30]. In a previous work [19], a custom-made pycnometer was designed and manufactured using a pressure transducer with a gauge pressure range of 0–350 kPa, an accuracy of 0.015% at full scale, and a block gauge with a 0.12 mm tolerance. The porosity range was 0.87–0.93, as shown in Table 1.

### 2.2. Test Section of Experiment

**Figure 2:**
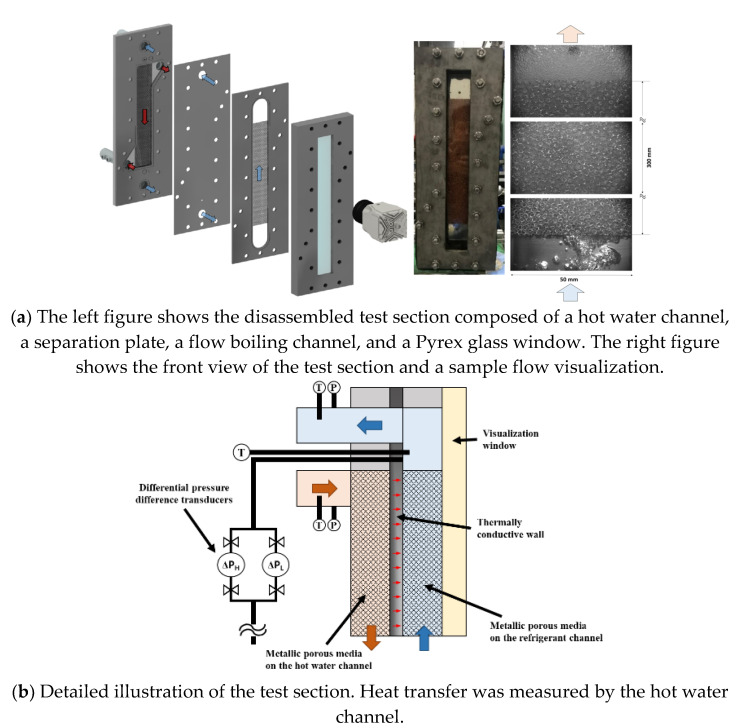
(**a**) Images of the parts and assembled test section and (**b**) detail cutting view of the test section.

A test module was prepared using one R245fa side boiling channel and one hot water channel, such as a general plate-type heat exchanger. The flow boiling channel was heated by counter flow hot water, and heat transfer occurred through a 2 mm thick stainless steel sheet, as shown in Figure 2a. The other side of the flow boiling channel was made from Pyrex glass, and the two-phase flow through the channel was visualized through the window. The R245fa flows vertically upward, which is the opposite direction that hot water naturally flows.

The channels were assembled with bolts, making it simple to clean the inside and switch to other samples. In these experiments, the nickel porous-foam sample was inserted into the hot-side channel to create a high-turbulence flow and uniform temperature distribution. Viton O-rings and proper seals were used between the visualization window and the plate to prevent leaks. The dimensions of the test area occupied by the metal foam were 300 by 50 mm, as shown in the right picture of Figure 2a.

The temperature and pressure differences through the two metal foam filled channels were measured by two pressure-difference transducers and two T-type thermocouples placed at both ends of the test sections. The thermocouple and pressure transducers were respectively located at the inlet and outlet ports. The detailed configurations and dimensions of the test section are shown in Figure 2b and Table 2.

### 2.3. Test Loop

**Figure 3:**
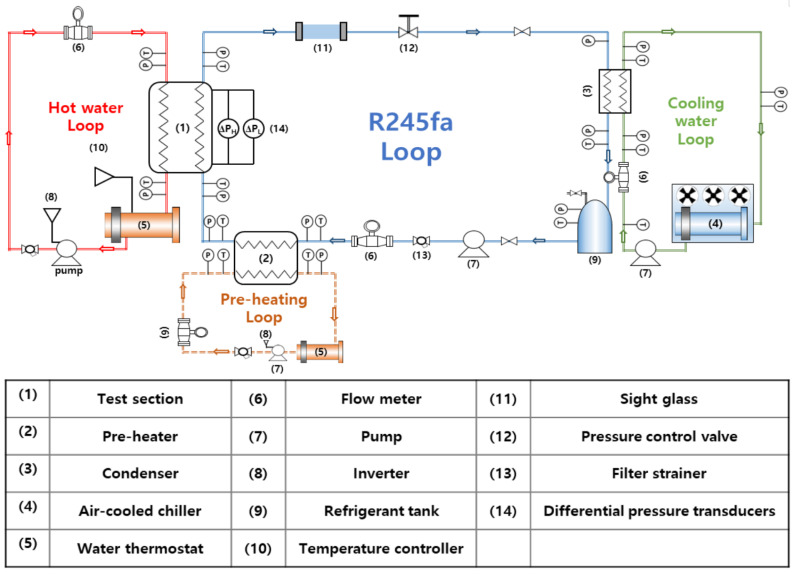
Diagram of the test loop and equipment/sensor positions.

As shown in Figure 3, the test loop consisted of four main loops: the refrigerant loop, the coolant loop, the hot source water loop, and the preheat water loop. The test channel was located between the hot water loop and the refrigerant loop. This loop was used to visualize the two-phase flow and measure the saturation boiling heat transfer of R245fa in the channel filled with porous metal foam. The refrigerant loop used R245fa as the working fluid, and a storage tank was located in front of the gear pump. In this system, the gear pump (Tuthill-DGS1) (Tuthill, Burr ridge, IL, USA)transports the refrigerant through the loop, and a gear-type flow meter (KEM-ZHA04) (KEM Küppers Elektromechanik GmbH, Karlsfeld, Germany)with a digital indicator, mounted downstream of the pump, is used to measure the refrigerant flowrate.

The hot water loop consists of a boiler with 30 kWe electric heater, a vertical multi-stage pump (LOWARA-1SV18F) (Xylem, Letchworth, UK), a PID temperature controller(Donghwa, Busan, Korea), and a geared flow meter(KEM Küppers Elektromechanik GmbH, Karlsfeld, Germany); the entire loop is insulated. A preheat loop was used to vary the inlet vapor quality of the refrigerant. The preheat loop consisted of a boiler with a partially built-in electric heater to prevent rapid evaporation in the tank. This loop can handle a wide range of heat capacities from 0 to 120 kW.

The vaporized refrigerant in the test section was cooled while passing through the condenser connected to the cooling water loop, which was operated by an air-cooled chiller, and moved to the refrigerant tank. During the experiment, the pressure and temperature values of the inlets and outlets of all components of the loop were measured using pressure transducers and T-type thermocouples. The loop was controlled by a pressure regulating valve located 1 m downstream from the outlet port of the test section so that the pressure measured at the inlet of the test section could be held constant at saturation pressure during operation of the test loop.

A LABVIEW system (National Instruments) was used for data acquisition. A custom LABVIEW program was written to receive all values for the flow, temperature, and pressure from the data acquisition system and convert the measured data into useful properties of water and R245fa at each point using the REFPROP 9.1 library. The operating conditions are given in Table 3. Nematollahi et al. used the same loop for their organic Rankine cycle power generation experiment, and the present operating conditions were in the same range as those for partial loading [4].

### 2.4. Experimental Procedures

To keep the R245fa saturated, the temperature of the hot water at the inlet of the preheat loop was adjusted to a predetermined temperature (70 °C). Then, the temperature and flow rate of the coolant in the sub-cooling loop were adjusted to keep the temperature of the coolant reservoir below 18 °C. After the circulation reached a steady state, the R245fa loop was activated. An inverter was installed on the refrigerant pump and the pressure control valve to control the mass-flow rate and saturation pressure, respectively. The inlet vapor quality of R245fa depended on the preheated water temperature and flow rate and could be changed to various values.

In the test section, the flow rate and inlet temperature were adjusted to decrease the heat-transfer rate due to the reverse flow of the hot water channel. Because the working pressure of the hot water channel can affect the HTC of the hot water channel, this pressure was kept constant via the pressure control valve during all experiments. In addition, the hot side was filled with 24 PPI nickel metal foam, which made the flow highly turbulent and kept the temperature change between the inlet and outlet below 1.5 K to maintain a constant wall temperature.

### 2.5. Patterned Configurations of Porous Media

To improve thermal efficiency, we investigated porous media partially filled with patterned configurations. In our previous study [29], the metal foam structure enhanced heat transfer significantly; however, an increased pressure drop was a drawback. To the best of our knowledge, there is no report on the usage of patterned metal foam for boiling heat transfer. An experimental investigation on the heat and fluid flow characteristics of different types of patterned configuration models with porous media was thus conducted. The boiling flow under three types of patterned models was examined and compared in a rectangular channel fully filled with the Cu-01 porous media. 

**Figure 4:**
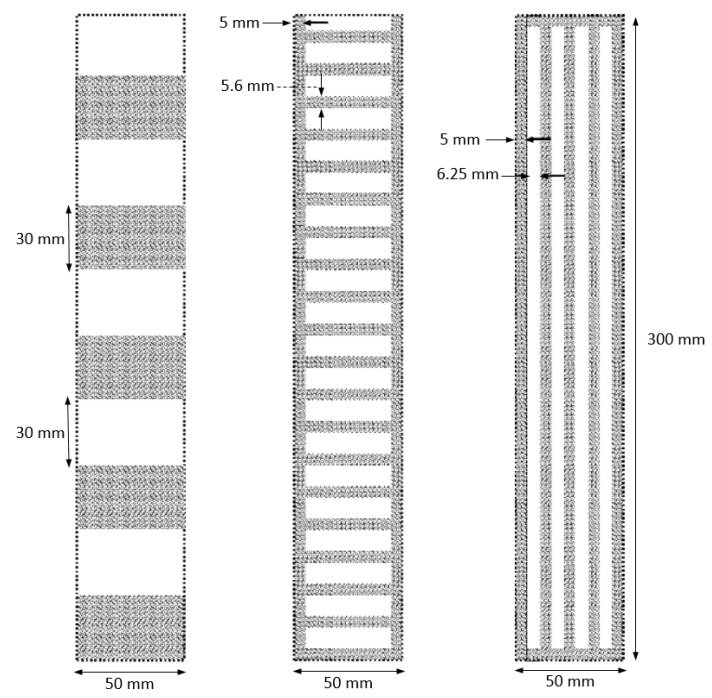
Dimension of the patterned models of porous media.

**Figure 5:**
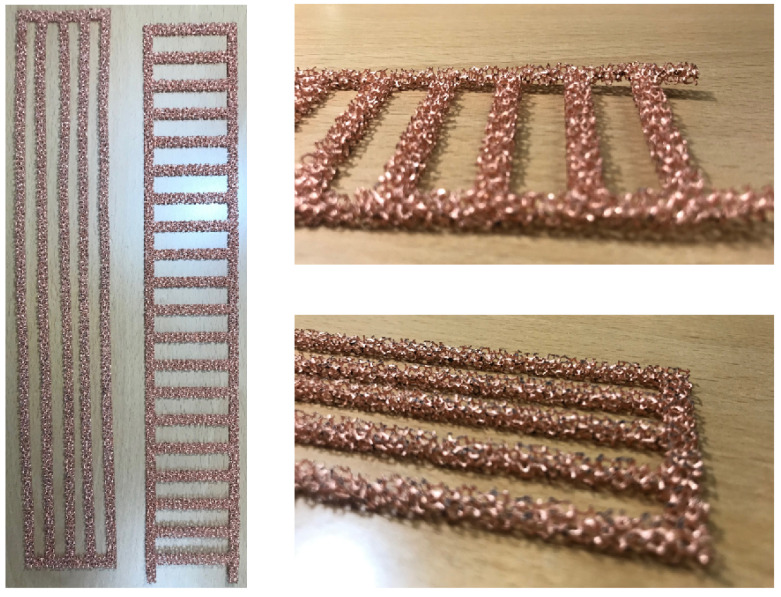
Images of the patterned models of the porous media.

In this study, two spanwise array models and one streamwise array model were designed, as shown in Figure 4. The spanwise array models included a sparse array model and dense array model, as shown in Figure 4 All of the patterned models occupied the same heat transfer area in as much as half of the full area of the channel. The patterned models are shown in Figure 5. The exact dimensions of the models are shown in Figure 4.

## 3. Results and Discussion

### 3.1. Flow Visualization

**Figure 6:**
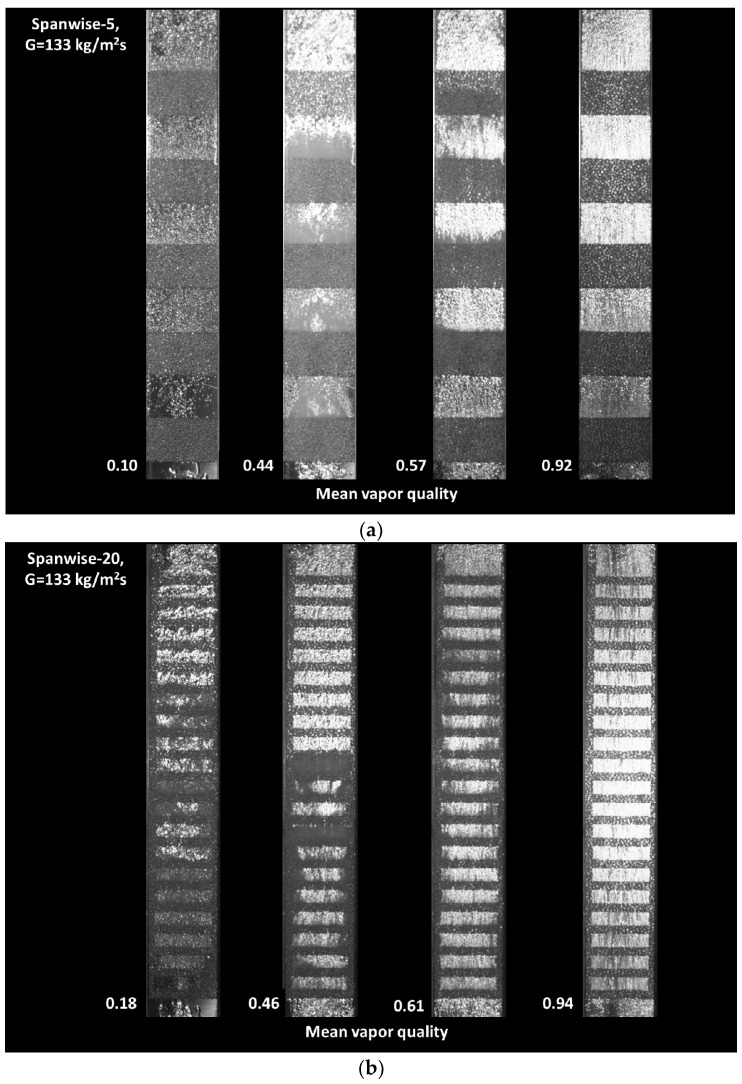

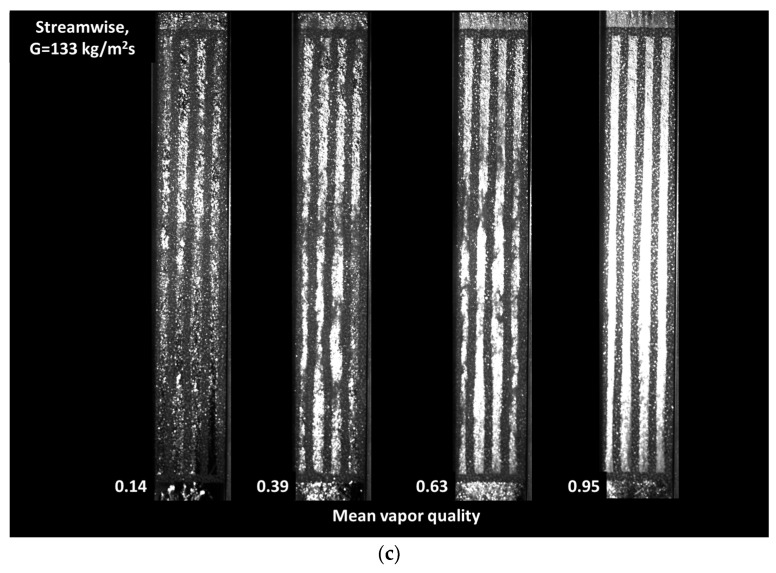
Visualizations of the boiling flow patterns of R245fa in (**a**) the spanwise-5 pattern model, (**b**) the spanwise-20 pattern model, and (**c**) the streamwise pattern model with 18 PPI copper porous media.

Figure 6 shows a visualization of the boiling flows in the patterned configurations. The inlet quality of the R245fa vapor was changed by controlling the preheat water temperature and flow rate. Using a high-speed camera (Phantom VEO410L) (Vision Research, Inc., Wayne, NJ, USA), flow images of different patterned metal foam insert configurations were taken under different vapor quality conditions with the same mass flow rate. Here, flow boiling is taken as a function of the average vapor quality at the inlet and outlet, as shown in Figure 6. In the metallic foam of the channel, the gas dominant phase is displayed in light gray, and the liquid dominant phase is dark gray. Here, four different flow patterns can be observed. The intermittent slug-fluctuating flow shows a mixed pattern of gray and a dark image. The fluctuating-annular flow shows wavy patterns due to the oscillatory or time-varying nature of the flow. A toroidal–fog flow pattern appeared after the stratified liquid film flowed over the surface, and the disappearing and entrained water droplets floated inside the channel. The fog streams pattern widened the dry areas and increased the small floating liquid particles.

Figure 6a shows the flow boiling patterns in the channel with five spanwise inserts. At a mean vapor quality of 0.10, intermittent slug-churn flow was observed at the inlet of the channel. The porous ligaments of metal foam disturbed the entrance of the big bubble due to a blockage effect. After passing through the first spanwise insert, the slug flow was destroyed by the metal foam, and tiny small bubbles were generated. However, the bubbly flow was not uniform. In the flow after the second, third, and fourth spanwise inserts, uniform bubbly flows were observed. After the final spanwise insert, the bubbly flow became intermittent because there was no metal foam downstream. 

At a mean vapor quality of 0.44, the flow boiling pattern assumed a churn-annular flow regime. Wavy patterns were clearly observable from the inlet to the fourth spanwise metal foam insert. After the fourth insert, a uniform bubbly flow was observed. At a mean vapor quality of 0.57, an annular-mist flow was observed. The bubbly flow appeared to be stable and uniform, but the local vapor quality increased with the flow passing the serial spanwise inserts. At a mean vapor quality of 0.92, the annular mist flow at the channel inlet became the mist-flow regime downstream. As the flow passed the spanwise insert, and the vapor quality improved. Dry-out regions were rarely found in the metal foam the channel.

Figure 6b shows the boiling flow patterns in the channel with 20 spanwise inserts. Under mean vapor quality of 0.18, slug-churn flow was observed at the inlet of the channel. After passing through the first spanwise insert, the slug bubble broke down and generated bubbles. However, the bubble distribution was non-uniform. In the flow between the second and seventh spanwise inserts, a uniform bubbly annular flow was observed. After the seventh spanwise insert, the churn-annular flow behaviors were intermittent up to the 15th spanwise insert. After the 16th insert, the annular flow became uniform due to the metal foam inserts. 

With mean vapor quality of 0.46, the flow boiling pattern assumed a churn-annular flow regime until the 12th spanwise insert. After the 12th insert, a uniform bubbly flow appeared, which happened earlier here than in the five spanwise inserts. Under vapor quality of 0.61, an annular-mist flow was observed throughout the whole channel, and weak wavy motion was observed on the side walls. Under quality conditions of 0.94, the annular mist flow at the channel inlet became a mist-flow regime downstream. The bubbles passing through the spanwise inserts were continuously broken down by the ligaments and coalesced in the cells. After the last metal foam insert, the bubbles coalesced again due to the wake flow that occurred in the metal foam ligaments.

Figure 6c shows the two-phase flow boiling patterns in the channel with five streamwise inserts. Here, two-phase flows move through four small channels between the streamwise metal foam inserts. At vapor quality of 0.14, intermittent slug-churn flow could be observed from the inlet to 1/4 of the whole channel length. The slug flow was destroyed by the porous metal foam when the flow moved spanwise. Under 0.39 vapor quality, the flow boiling pattern assumed a churn-annular flow regime. Wavy patterns were observed but were attenuated by the porous side walls. Under 0.63 mean-vapor quality conditions, the annular-mist flow was observed throughout the whole channel. A coalescence of bubbles in the narrow channels was also observed. Under 0.95 mean vapor quality, only annular mist flow was observed throughout the whole channel.

### 3.2. Heat Transfer

The vapor quality of a fluid boiling at saturation can be calculated indirectly based on the heat flux of a single-phase water flow on a hot surface. Because the R245fa was heated to a saturation state before entering the test section, the vapor quality at the outlet of the preheater was estimated. At this stage, it can be assumed that the outlet vapor quality of the preheater is equal to the inlet vapor quality of the test section because the distance is short between the outlet of the preheater and the inlet of the test section. The inlet vapor quality of the test section was calculated using the following equation [31]:

(1) $$x_{pre,o}=x_{i}=\frac{1}{i_{c,lv}}\left[\frac{Q_{pre}}{m_{c}}-\left(i_{c,sat,l}-i_{c,pre,i}\right)\right]$$

where $i_{lv}$ is the latent heat of the refrigerant equal to the heat-transfer rate of water in the preheater, which was calculated from

(2) $$Q_{pre}=m_{pre}\left(i_{pre,i}-i_{pre,o}\right)$$

In this study, the thermodynamic equilibrium quality was adopted to express the vapor quality under saturated conditions based on thermodynamic properties:

(3) $$x=\frac{i-i_{l}}{i_{lv}}$$

Thermodynamic equilibrium quality equal to zero indicates a saturated liquid, while a value of one indicates saturated vapor. Moreover, x < 0 indicates a subcooled liquid state and x > 1 indicates superheated vapor.

The change in the vapor quality of the refrigerant through the test section, Δx, can be derived from the heat-transfer rate of the hot water channel in the test section [32]:

(4) $$\Delta x=x_{0}-x_{i}=\frac{Q_{h}}{m_{c}i_{lv}}$$

Here, the overall HTC for the saturated flow boiling of R245fa in the test section is the same as that of the single-phase water flow except for the change in the log-mean temperature difference for the saturation of R245fa:

(5) $$U=\frac{Q_{w}}{HTA\cdot \Delta T_{LMTD-sat}}\;$$

where *HTA* is the effective surface area of heat transfer, and $\Delta T_{LMTD-sat}$ is the log-mean temperature difference in a counter-flow heat exchanger [33]:

(6) $$\Delta T_{LMTD}=\frac{\Delta T_{1,sat}-\Delta T_{2,sat}}{\ln\frac{\Delta T_{1,sat}}{\Delta T_{2,sat}}}$$

Under saturated boiling conditions, the temperature differences are defined as

(7) $$\Delta T_{1,sat}=T_{h,i}-T_{c,o,sat}$$

(8) $$\Delta T_{2,sat}=T_{h,o}-T_{c,i,sat}$$

Lastly, the HTC for the saturated boiling of R245fa is obtained as follows:

(9) $$\frac{1}{h_{c,tp}}=\frac{1}{U}-\frac{1}{h_{h}}-\frac{\delta }{k_{w}}$$

**Figure 7:**
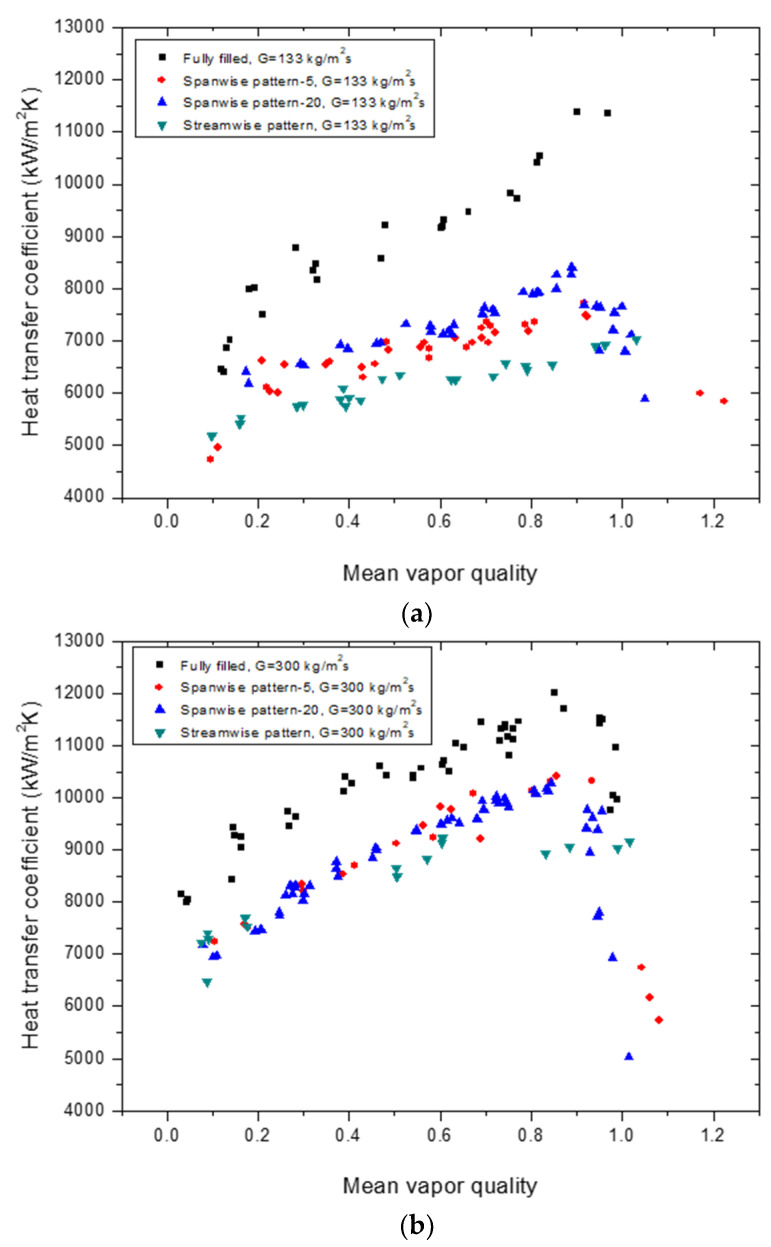
Comparison of the boiling HTC per unit mass flux at (**a**) G = 133 kg/m^2^s and (**b**) G = 200 kg/m^2^s for different patterned models of porous media with a fully filled porous media channel.

The boiling HTC values of three different types of patterned configuration models were compared with those of a fully filled channel at G = 133 and 300 kg/m^2^s, as shown in Figure 7. As the mean vapor quality increased, the HTC tended to increase, and, as expected, the fully filled channel had the highest thermal performance. The HTC of the dense-array spanwise pattern model (spanwise pattern-20) was higher than that of the sparse array model (spanwise pattern-5). The streamwise pattern model had the lowest HTC value at a lower mass flux (G = 133 kg/m^2^s), as shown in Figure 7a. However, as shown in Figure 7b, at a higher flow rate (G = 300 kg/m^2^s), the deviation of HTC from the two other patterned models was reduced, giving all three models similar HTC values. 

The trend of the boiling HTC with respect to the mean vapor quality was mostly similar to that of the fully filled channel. However, the streamwise pattern model was exceptional. No rapid increase of HTC was observed in the high vapor quality region. On the other hand, regardless of flow conditions, both graphs show rapid drops of HTC when the mean vapor quality value was close to 1 ($x_{m}\approx 1$). According to Kim et al. [29], this happens because the fluid starts to dry out and becomes completely vaporized.

An uncertainty analysis was carried out using the procedure suggested by Moffat [34]. The uncertainty of the length, width, and height of the test section was estimated to be ±0.1 mm. The K-type thermocouples had uncertainty of ±0.20%, the pressure transducers had uncertainty of ±4.36%, and the flowmeter had uncertainty of ±4.72%. The system error and random error values were then calculated for the number of samples and the standard deviation of the measured data. The results indicated that the heat-transfer rate had ±6.43% uncertainty in a 95% confidence range. Based on these uncertainties, the HTC has an uncertainty of ±7.98%. Therefore, the uncertainty of this experiment is acceptable compared to that in previous studies.

### 3.3. Pressure Drop

The total pressure drop was measured between the inlet and outlet of the channel section filled with the metallic porous specimen. The frictional pressure drop was obtained by subtracting the acceleration pressure drop and gravitational pressure drop from the total pressure drop:

(10) $$\Delta P_{total}=\Delta P_{f}+\Delta P_{a}+\Delta P_{g}$$

where the acceleration pressure drop is calculated by a separate model [35]:

(11) $$\Delta P_{a}=-G^{2}v_{l}\int_{in}^{out}\left[\frac{x^{2}}{\alpha }\left(\frac{v_{g}}{v_{l}}\right)+\frac{{\left(1-x\right)}^{2}}{1-\alpha }\right]dz$$

The cross-sectional void fraction, α, is calculated using the Steiner version of the Rouhani–Axelsson drift flux model [36]:

(12) $$\alpha =\frac{x}{\rho_{v}}{\left[\left(1+0.12\left(1-x\right)\right)\left(\frac{x}{\rho_{v}}+\frac{1-x}{\rho_{l}}\right)+\frac{1.18\left(1-x\right)[g\sigma {\left(\rho_{l}-\rho_{v}\right)}^{1/4}}{G\rho_{l}^{0.5}}\right]}^{-1}$$

The gravitational pressure drop under the vertical configuration of the test section is expressed as follows [35]:

(13) $$\Delta P_{g}=\frac{Lgsin\theta }{x}\int_{x^{in}}^{x_{out}}\left[\rho_{g}\alpha +\rho_{l}\left(1-\alpha \right)\right]dx$$

The two-phase Fanning friction factor is calculated as follows:

(14) $$f_{tp}=\frac{\Delta p_{tp,f}D_{h}\rho_{m}}{2G^{2}L}$$

where $\rho_{m}$ is the mean density of the fluid, which is expressed as

(15) $$\frac{1}{\rho_{m}}=\left[\frac{x_{m}}{\rho_{g}}+\frac{\left(1-x\right)}{\rho_{l}}\right]$$

**Figure 8:**
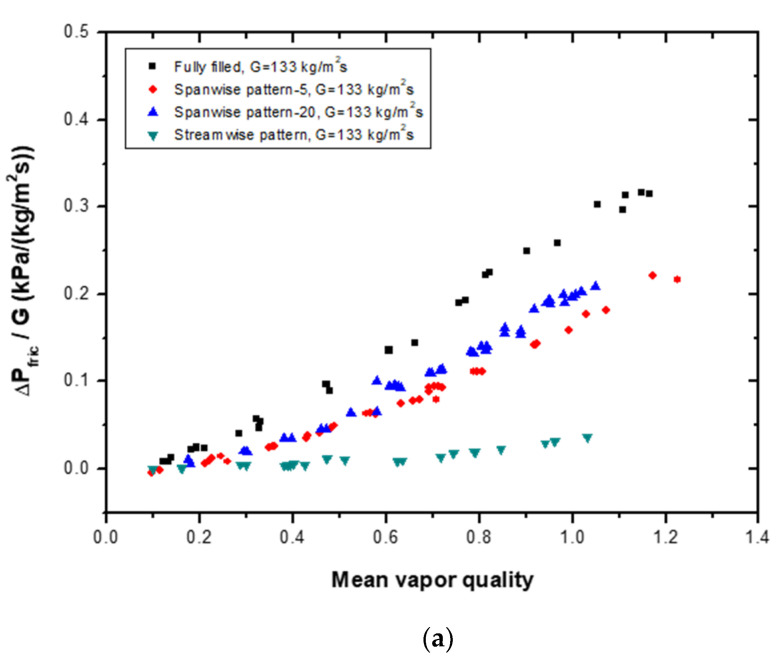

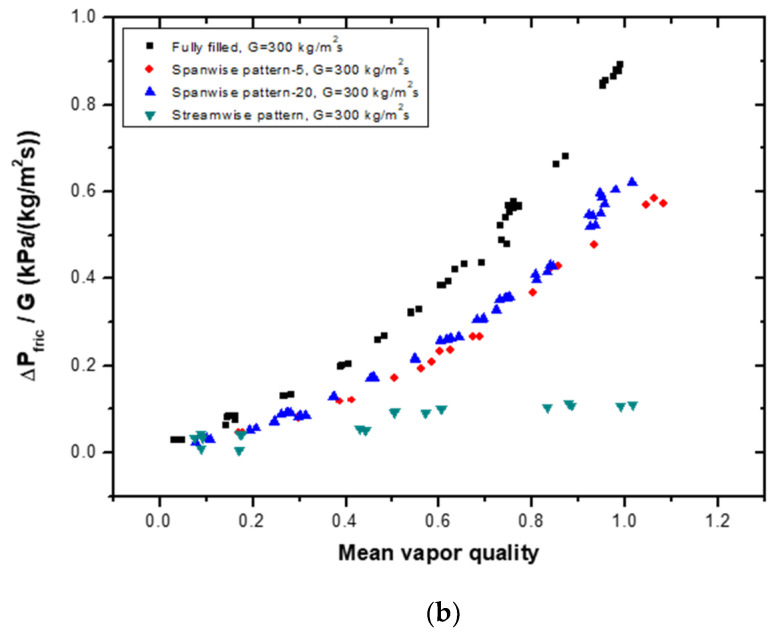
Comparison of the two-phase frictional pressure drop per unit mass flux at (**a**) G = 133 kg/m^2^s and (**b**) G = 200 kg/m^2^s for different patterned models of porous media with a fully filled porous media channel.

The two-phase frictional pressure drop per unit mass flux of different types of patterned models was compared with that of a fully filled channel, as shown in Figure 8. The fully filled channel understandably featured the highest pressure drop. Between the two spanwise pattern models, the sparse model had a slightly lower pressure loss than the dense model. Notably, the streamwise pattern model also had a much lower pressure drop than that of the other models. The pressure loss of the streamwise pattern model was up to 1/8 times lower than that of the fully filled channel at a lower mass flux (Figure 8a). Under a higher mass flux, the pressure loss in the streamwise metal foam insert channel was much less than that of the other channels.

**Figure 9:**
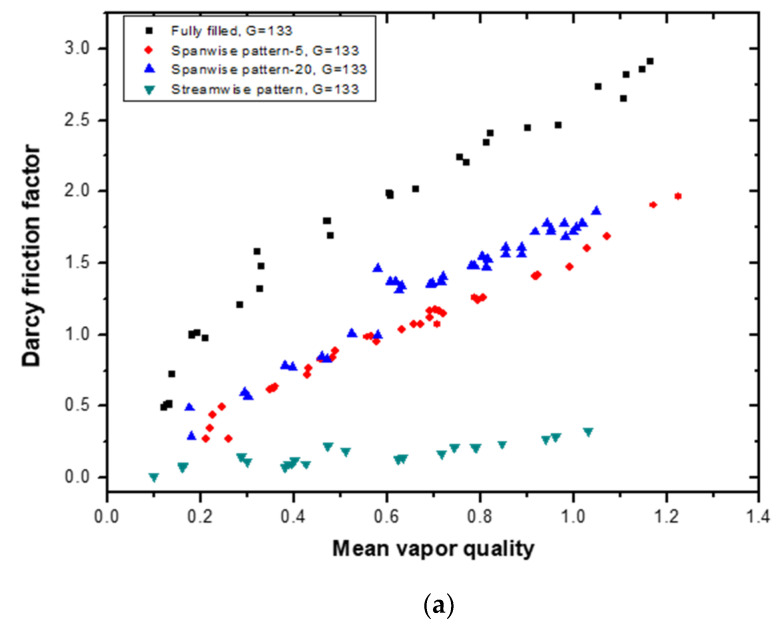

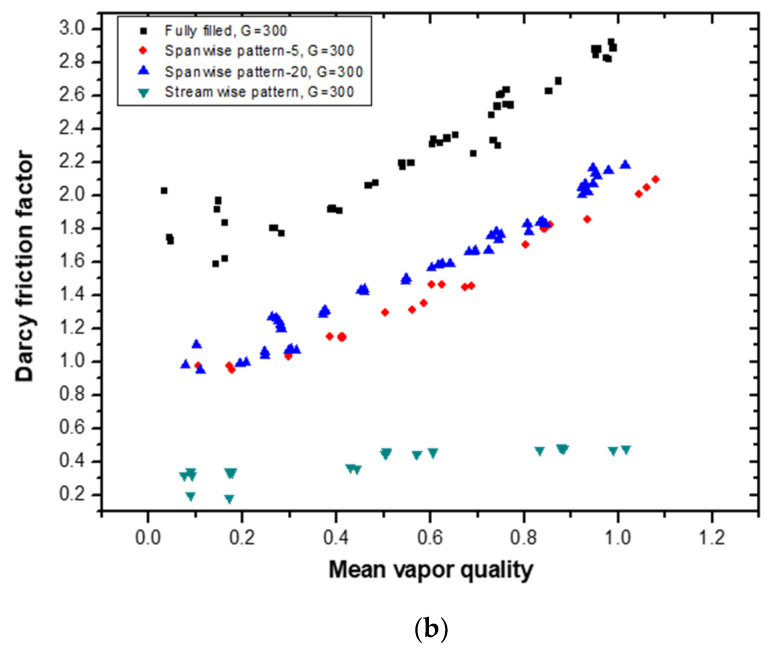
Comparison of the Darcy friction factor per unit mass flux at (**a**) G = 133 kg/m^2^s and (**b**) G = 200 kg/m^2^s for different patterned models of porous media with a fully filled porous media channel.

The Darcy friction factor can be obtained using the following frictional pressure drop:

(16) $$f_{D}=\frac{2D_{h}\Delta P_{fric}}{\rho {\langle u\rangle }^{2}L}$$

where *ρ* is the fluid density and *u* is the channel inlet velocity of the fluid. The Darcy friction factor of the patterned models has a linearly increasing trend with respect to the vapor quality, as shown in Figure 9. The fully filled channel has the highest friction factor value (up to 2.96), and the streamwise pattern model has the lowest value. The maximum is 0.49 under these experimental conditions. The dense array model has a slightly higher friction factor than the sparse array model.

### 3.4. Goodness Factors

The performance of heat-exchange surfaces composed of different idealized passage geometries can be contrasted through the following two complementary measures: the flow area’s goodness factor and the core volume’s goodness factor. The ratio of the Colburn *j* factor to the Fanning friction factor, *f*, compared to the Reynolds number is generally known as the flow area’s goodness factor, which was suggested by Shah and London [37]. The flow area’s goodness factor is expressed using Equation (17):

(17) $$G_{a}=\frac{j}{f}=\frac{{\bar{Nu}}_{tp}Pr_{m}^{-1/3}}{fRe_{eq}}$$

The Fanning friction factor is conventionally given as

(18) $$f=\frac{\Delta P\rho D_{h}}{2LG^{2}}$$

The Stanton number and Colburn j factor are:

(19) $$\mathrm{St}=\frac{{\bar{Nu}}_{tp}}{Re_{eq}Pr_{m}}\;$$

(20) $$j=StPr_{m}^{2/3}$$

The volume’s goodness factor is defined as:

(21) $$G_{v}=\frac{St}{f^{\frac{1}{3}}}$$

**Figure 10:**
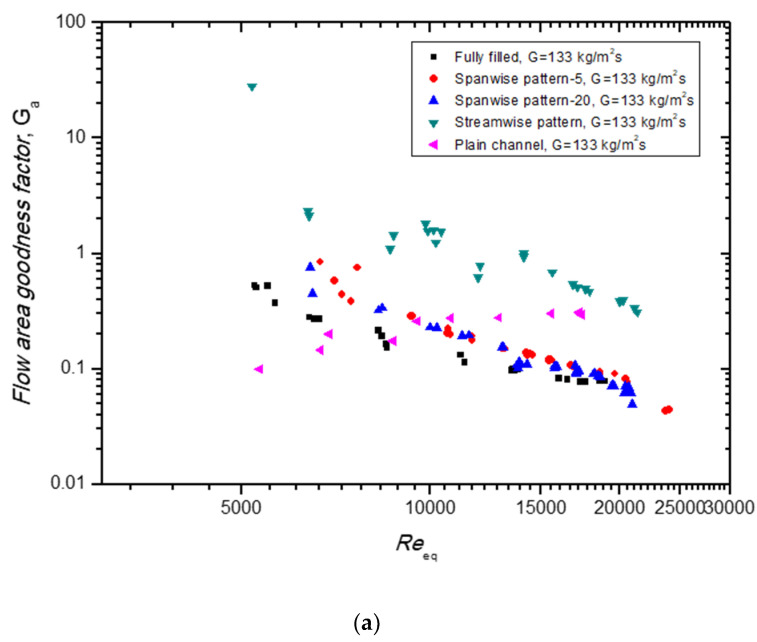

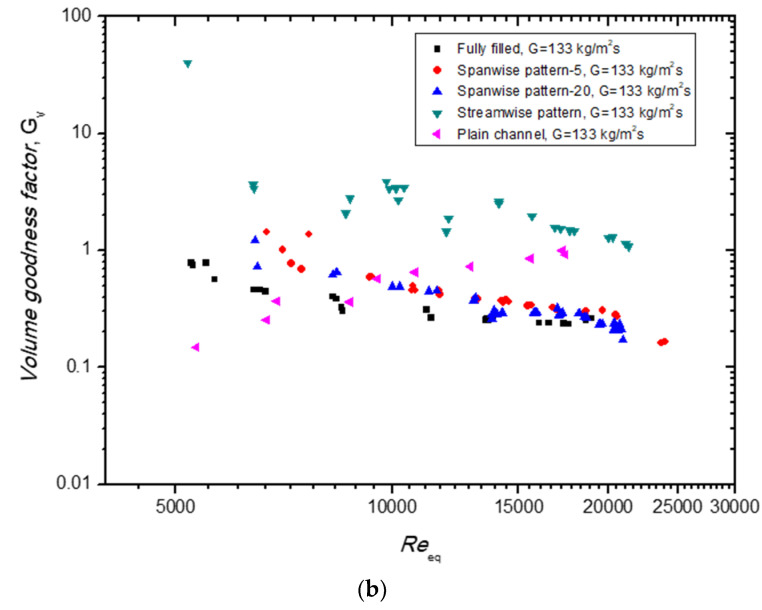
Comparison of (**a**) flow area’s goodness factor and (**b**) volume’s goodness factor of different patterned models of porous media with the fully filled porous media channel and plain channel at G = 133 kg/m^2^s.

Figure 10 presents the area’s goodness factor and the volume’s goodness factor for the patterned models compared to those of the fully filled channel and the plain channel according to the equivalent Reynolds numbers (*Re_eq_*). As shown in Figure 10a, the area’s goodness factor (*G_a_*) of the streamwise pattern model had the highest value among the models. The *G_a_* was also higher than that of the plain channel. The goodness factor of the porous media channel presented a decreasing trend, along with *Re_eq_*, due to the strong pressure-loss properties, while the plain channel’s goodness factor increased. Similar to the area’s goodness factor, the volume’s goodness factor (*G_v_*) of the streamwise pattern model was the highest among the porous models and the plain channel.

**Figure 11:**
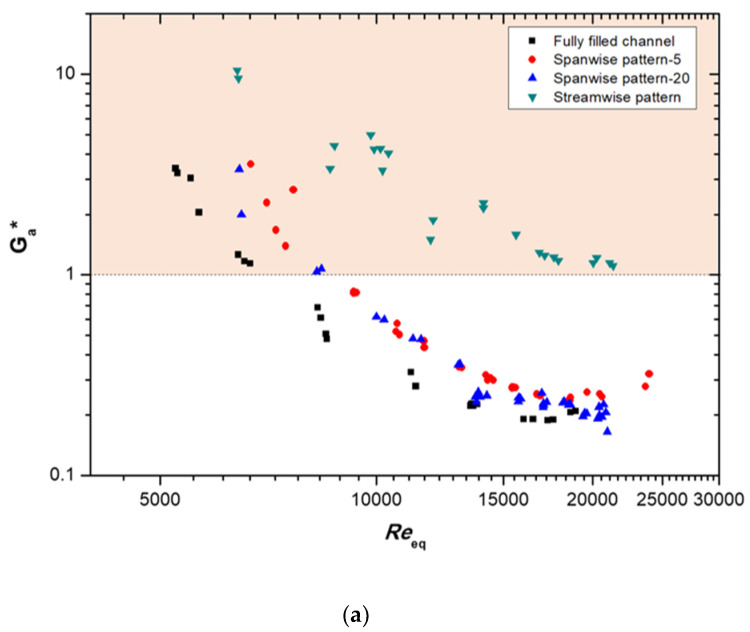

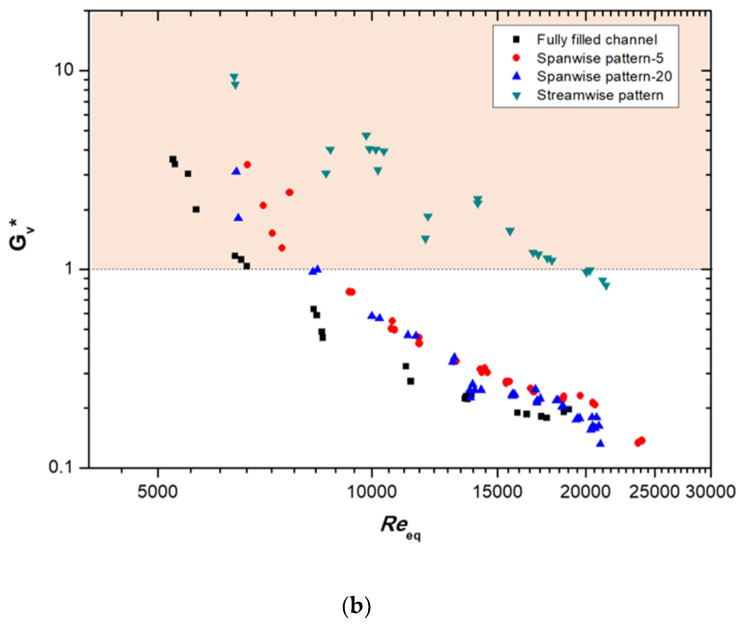
Comparison of (**a**) the ratio of the flow area’s goodness factor and (**b**) the ratio of the volume’s goodness factor between the porous patterned models and the plain channel at G = 133 kg/m^2^s.

The ratios of the goodness factors of the porous patterned configuration models and those of the plain channel are compared in Figure 11. The goodness factor ratio, G*, is expressed using Equation (22):

(22) $$G^{*}=\frac{G_{\left(Prous\;model\right)}}{G_{\left(Plain\;channel\right)}}$$

The goodness factor ratios are higher than 1 at a low *Re_eq_*. These ratios became lower than 1 at *Re_eq_* > 6700 in the fully filled channel with Cu-01 and at *Re_eq_* > 8300 in the spanwise patterned porous channel. However, the streamwise patterned porous channel featured goodness factor ratios higher than 1 throughout almost all experimental cases. At the lowest *Re_eq_* of 6400, the volume’s goodness factor ratio of this model increased to 9.4, and the flow area’s goodness factor ratio increased up to 10.5; these values fell as the *Re_eq_* increased. As a result, we propose that a metallic porous-media heat exchanger could be optimized for specific operating conditions by properly designing the media’s patterned configuration.

## 4. Conclusions

This study explored the high-speed visualization of the flow boiling of R245fa in a rectangular channel filled with open-cell metal foam inserts, as well as the boiling heat transfer and frictional pressure drop of a two-phase vertical up-flow inside the channel. We conducted an experimental investigation of the heat and fluid flow characteristics in a fully filled channel and several kinds of patterned configuration models. The HTC tended to increase with the mean vapor quality, and, as expected, the fully filled channel had the highest thermal performance. The dense-array spanwise pattern model’s HTC was higher than that of the sparse-array model, and the streamwise pattern model had the lowest HTC value at a low mass flux (G = 133 kg/m^2^s). However, at a higher flow (G = 300 kg/m^2^s), the deviation of HTC among the three different patterned models was reduced, as all model’s had similar HTCs.

Notably, the streamwise pattern model presented a very low pressure drop compared to the other models. The pressure loss value was up to 1/8 times lower than that of the fully filled channel. The flow area’s goodness factor (Ga) and the core volume’s goodness factor (Gv) were then used to compare the performance of heat-exchange surfaces composed of different arbitrary passage channels. Based on our results, metallic porous-media heat exchangers could be optimized for specific operating conditions (e.g., mean vapor quality, flow mass flux, etc.) by properly designing the patterned configuration of the porous media.

## Figures and Tables

**Table 1:** Geometric parameters of specimens.

Materials	d_p_ (mm)	d_f_ (mm)	d_w_ (mm)	ε	PPI	d_w_/d_f_
Cu	1.006 ± 0.154	0.384 ± 0.155	1.390 ± 0.155	0.893 ± 0.019	18	3.62
Ni	0.808 ± 0.165	0.261 ± 0.026	1.069 ± 0.167	0.922 ± 0.011	24	4.10

**Table 2:** Dimensions of the test section and metal foam.

Parameters	Dimensions
Width of channel (mm)	50
Length from port to port (mm)	390
Height of channel (mm)	5
D_port_ (mm)	20
Thickness of separation plate (mm)	2
Size if metal foam W × L × T (mm)	50 × 300 × 5

**Table 3:** Experimental conditions.

Parameters	Values
Metal foam specimens (water/R245fa)	Nickel-24 PPI/Copper-18 PPI
Working fluids (hot/cold)	Water/R245fa
Mass flux (R245fa) (kg/m^2^s)	133–300
Inlet temperature (preheat) (°C)	70
Inlet temperature (hot water) (°C)	73
Saturation pressure (bar)	5.9
Inlet vapor quality	0.05–0.99

## Data Availability

The data presented in this study are available on request from the corresponding author.

## Nomenclature

*Pr*	Prandtl number [$c_{p}\;\mu /k$]
*Nu*	Nusselt number [h$D_{h}/k_{l}$]
*Re*	Reynolds number [G$D_{h}/\mu$]
$Re_{eq}$	Equivalent Reynolds number [$G_{eq}D_{h}/\mu_{l}$]
*G*	Mass flux [kg/m^2^s]
*G_a_*	The flow area’s goodness factor
*G_v_*	The volume’s goodness factor
*G**	Goodness factor ratio
*PPI*	Pores per inch, [-]
*HTA*	Effective heat transfer coefficient [W/m^2^K]
*HTC*	Heat transfer coefficient [W/m^2^K]
*U*	Overall heat transfer coefficient [W/m^2^K]
*Q*	Heat-transfer rate [kW]
$\Delta T_{LMTD}$	Log-mean temperature difference, [°C]
$\Delta P_{total}$	Total pressure drop [Pa]
$\Delta P_{fric}$	Frictional pressure drop [Pa]
$\Delta P_{g}$	Gravitational pressure drop [Pa]
$D_{h}$	Channel hydraulic diameter [m]
L	Channel length [m]
L_e_	Equivalent fin length [m]
H	Channel height [m]
W	Channel width [m]
c_p_	Specific heat [kJ/kg K]
m	Mass flow rate [kg/s]
u	Inlet velocity [m/s]
f	Fanning friction factor [-]
j	Coliburn j factor [-]
St	Staton number [-]
x	Vapor quality [-]
i	Internal energy [-]
v	Specific volume [m^−3^]
g	Gravitational acceleration [m/s^2^]
k	Heat conductiviy coefficient [W/m K]
T	Temperature [°C]

## Greek Symbols

α	Void fraction [-]
δ	Channel wall thickness [m]
ε	Porosity [-]
ρ	Density [kg/m^3^]
μ	Dynamic viscosity [kg/m s]
σ	Surface tension [N/m]
θ	Angle between flow direction and gravity vector [-]

## Subscripts

c	Cold side
h	Hot side
i	Inlet
o	Outlet
eff	Effective
m	Mean value
avg	Average value
f	Fluid
s	Solid
w	Channel wall
tp	Two-phase
pre	Preheater
eva	Evaporator
l	Liquid
v	Vapor
g	Saturated vapor
Sat	Saturation
